# Ring Finger Protein 34 (RNF34) as a Prognostic Biomarker for Clear Cell Renal Cell Carcinoma

**DOI:** 10.7759/cureus.53038

**Published:** 2024-01-27

**Authors:** Johannes Stein, Niklas Klümper, Pirmin Zöhrer, Thomas Büttner, Philipp Krausewitz, Manuel Ritter, Glen Kristiansen, Marieta Toma, Jörg Ellinger, Alexander Cox

**Affiliations:** 1 Urology, University Hospital Bonn, University of Bonn, Bonn, DEU; 2 Center for Integrated Oncology Cologne/Bonn, University Hospital Bonn, University of Bonn, Bonn, DEU; 3 Pathology, University Hospital Bonn, University of Bonn, Bonn, DEU

**Keywords:** clear cell renal cell carcinoma, tcga, immunohistochemistry, biomarker, rnf34, renal cell carcinoma

## Abstract

Introduction: Ring finger proteins play pivotal roles in diverse cellular processes and are implicated in contribution to cancer. Ring finger protein 34 (RNF34) has antiapoptotic and oncogenic properties. RNF34 is upregulated during carcinogenesis and tumor progression in the colorectal adenoma-carcinoma sequence and was already described to mediate chemoresistance. In clear cell renal cell carcinoma (ccRCC), however, the role and expression patterns of RNF34 are unknown.

Methods: First, we investigated the association of RNF34 mRNA expression with clinicopathological parameters and survival using data obtained from The Cancer Genome Atlas (TCGA) ccRCC cohort (N = 533). To assess RNA34 protein expression, we performed immunohistochemical (IHC) staining of an established ccRCC cohort (University of Bonn) in a tissue microarray (TMA) format. This validation cohort contains 109 primary ccRCC samples. IHC data were associated with clinicopathological parameters and overall survival (Kaplan-Meier analysis). Adjustment for covariables was done using the Cox regression model.

Results: RNF34 expression is correlated with adverse clinicopathological parameters. Survival analysis revealed an association between RNF34 expression and shortened survival. Cox regression analysis confirmed RNF34 expression as an independent prognostic parameter.

Conclusion: Our study provides evidence for RNF34 as a prognostic biomarker in ccRCC and points toward a major role of this protein in renal cell carcinoma carcinogenesis.

## Introduction

Renal cell carcinoma (RCC) is the most prevalent malignancy of the kidney in adults with an increasing incidence [1]. The predominant histologic subtype is clear cell RCC (ccRCC), which accounts for approximately 80% of tumors, followed by papillary and chromophobe RCC [2]. Localized disease is curable in the long term by surgical resection; the metastatic stage implies a shortened five-year survival of approximately 12% and requires drug tumor therapy [3]. Despite the expansion of the therapeutic armamentarium against metastatic RCC by the addition of immune checkpoint inhibitors to the widespread use of tyrosine kinase inhibitors, long-term response and significant survival benefits are far from evident in all patients [4,5]. For this reason, it is tremendously important to identify new diagnostic and therapeutic molecular biomarkers that predict tumor aggressiveness and expand the therapeutic arsenal toward molecular precision oncology.

The ring finger protein 34 (RNF34) is an E3 ubiquitin-protein ligase that is involved in the regulation of various cellular processes through ubiquitin-mediated proteasomal degradation of various proteins. RNF34 has been identified to exhibit antiapoptotic properties in part by ubiquitinating caspases 8 and 10 and thereby inducing their proteasomal degradation, which ultimately inhibits death receptor-mediated apoptosis [6]. Due to these oncogenic features, RNF34 plays a crucial role in colorectal carcinogenesis, in which its overexpression exerts a negative impact on the cell death signaling pathway [7]. By participating in the proteasomal degradation of the tumor suppressor protein p54, RNF34 provides further oncogenic potential [8]. The antiapoptotic function brought RNF34 into the focus of cancer research. Thus, RNF34 has also been shown to be preferentially expressed in esophageal and gastric cancers [9]. However, the role of RNF34 in the development and progression of RCC as well as its implication as a biomarker remains to be elucidated. Hence, our study aimed to comprehensively investigate RNF34 in ccRCC.

## Materials and methods

Transcriptome data assembly

The log2 transformed RNA sequencing data, generated using Illumina HiSeq (Illumina, Inc., San Diego, CA) technology and publicly available through The Cancer Genome Atlas (TCGA) Research Network, were downloaded from the UCSC Xena browser (http://xena.ucsc.edu). The RCC TCGA (http://cancergenome.nih.gov/) cohort comprised in total of 606 samples consisting of 533 tumor samples and 73 normal adjacent tissue (NAT), respectively (clinicopathological parameters are shown in Table 1). All patients registered in the TCGA Research Network had signed informed consent before registration. Clinicopathological data were accessible for the whole cohort. For the majority of the RCC cohort, clinical follow-up and overall survival (OS) data were available (n = 533; mean follow-up period = 3.7 years; range = 0-12.4 years). The median of the mRNA expression data was used as a cut-off value for dichotomization for survival analyses.

**Table 1:** Clinicopathological data of TCGA and IHC cohorts. TCGA: The Cancer Genome Atlas; IHC: immunohistochemical; NA: not applicable.

Parameter	TCGA	IHC
	n = 533 (%)	n = 109 (%)
Sex		
Male	345 (64.7)	74 (67.9)
Female	188 (35.3)	35 (32.1)
Age		
Mean	60.6	63.2
Min-max	26-90	33-84
Pathological stage		
pT1	271	41
pT2	68	22
pT3	183	44
pT4	11	2
Lymph node metastasis	16	9
Distant metastasis	79	16
Fuhrman grading		
G1	14	34
G2	229	72
G3	206	3
G4	76	0
NA	8	-

Immunohistochemistry

We analyzed RNF34 protein expression in a ccRCC paraffin-embedded kidney tissue microarray (TMA) cohort. TMAs were constructed as previously described [10,11]. In brief, in this study, 109 patients who had undergone (partial) nephrectomy at the University Hospital of Bonn between 1994 and 2008 were included (clinicopathological parameters are shown in Table 1). For each patient, three tissue cores of paraffin-embedded kidney tissue were collected. Tissues that were insufficient or did not contain carcinoma were excluded. The collected tissues were cut into 5 µm slices, mounted on slides, and stained using the specific polyclonal rabbit anti-RNF34 antibody in a 1:40 dilution (SAB2900260, Sigma-Aldrich, St. Louis, AZ). The staining process was carried out at the Ventana Benchmark automated staining system (Ventana Medical System, Tucson, AZ), as described before [10,11]. The staining quality and specificity were confirmed by an experienced uropathologist (MT). The slides were counterstained and digitalized. Three observers (NK, JS, and MT) independently evaluated the intensity of the RNF34 staining and assigned a score of 0 to 3 (negative, weak, moderate, or strong). Membranous, cytoplasmic as well as nuclear staining was assessed separately. In case of disagreement, the ratings were discussed and a consensus was reached. In the survival analysis, the cut-off describing positive expression was set at ≥1.

Statistical analyses

TCGA transcriptome sequencing data (RNA-Seq v2) were generated by Illumina HiSeq. Data were downloaded from the UCSC Xena browser (http://xena.ucsc.edu). Statistical analyses were performed using Microsoft Excel (Microsoft Corporation, Redmond, WA) and SPSS version 27.0 (IBM Corp., Armonk, NY). The Mann-Whitney U test or Kruskal-Wallis test was used to compare groups in terms of continuous variables. The chi-square test was employed to examine the associations between categorical variables and to assess the significance of any observed relationships. Survival analysis was carried out using Kaplan-Meier estimate curves and log-rank tests. Cox regression models were used for adjustment for covariables. GraphPad Prism (GraphPad Software, San Diego, CA) was used for visualization of the data.

## Results

mRNA analysis: high mRNA levels were correlated with advanced tumor stages and survival

**Figure 1:**
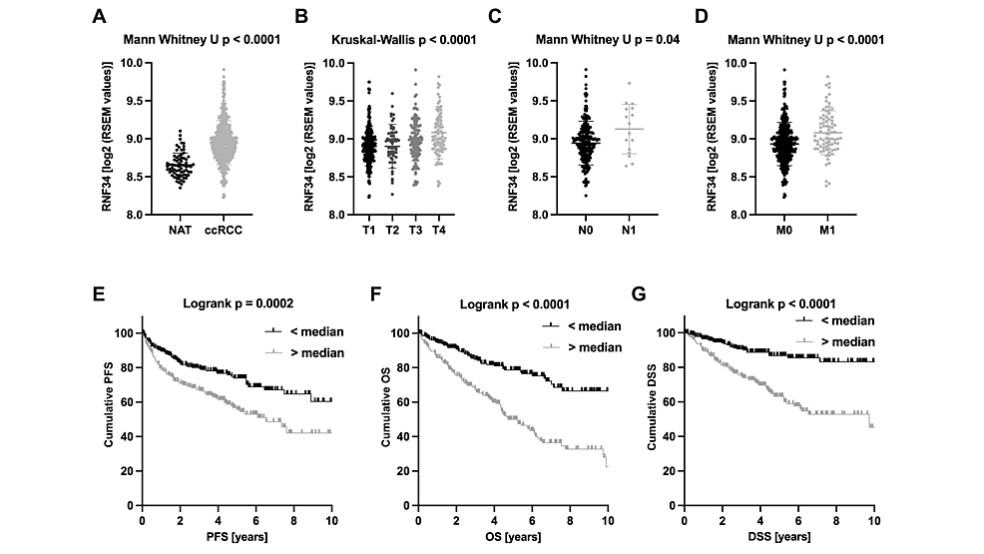
Transcriptome sequencing data on RNF34 in ccRCC obtained from TCGA. (A) RNF34 mRNA expression is significantly higher in RCC compared to NAT (p < 0.001). (B) Sequentially higher expression levels were found in more advanced tumor stages (p < 0.001). (C, D) Increased mRNA levels of RNF34 are associated with nodal-positive and metastatic disease (p = 0.04 and p < 0.001). (E-G) RNF34 mRNA expression was associated with a shortened progression-free, overall, and disease-specific survival (each Log-rank p < 0.001). Expression was dichotomized using the median as the cut-off value. p < 0.05 was considered as significant. RCC: renal cell carcinoma; ccRCC: clear cell renal cell carcinoma; TCGA: The Cancer Genome Atlas; NAT: normal adjacent tissue; PFS: progression-free survival; OS: overall survival; DSS: disease-specific survival.

Analyses of TCGA transcriptome data of RCC tissues showed that RNF34 mRNA is significantly overexpressed in RCC versus normal tissue adjacent to the tumor (NAT; p < 0.01; Figure 1A). Lower mRNA levels were found in tissues from patients with M0 and N0 stages than in patients with distant or lymphogenic metastases indicating an association with tumor aggressiveness (p < 0.01 and p = 0.041; Figures 1C, 1D). Furthermore, RNF34 mRNA expression correlates with pathological T stage and grading, as higher levels were found in more advanced stages and poorly differentiated tumors, respectively (each p < 0.01; Figure 1B). In accordance with these data, RNF34 mRNA overexpression was associated with unfavorable overall survival (OS), cancer-specific survival (CSS), and progression-free survival (PFS) in RCC as evaluated by Kaplan-Meier estimates and significant log-rank p-values (each log-rank p < 0.01; Figures 1E-1G).

The multivariate Cox regression analysis, including TNM stage parameters and grading, additionally showed that increased RNF34 expression was a significant and independent predictor of shortened OS and CSS in patients with ccRCC (OS: hazard ratio (HR) = 2.655, 95% CI = 1.64-4.30; CSS: HR = 2.534, 95% CI = 1.34-4.78, each p < 0.01; Tables 2, 3). An independent impact on PFS could not be revealed for RNF34 mRNA expression (univariate Cox regression analysis; PFS: HR = 1.815, 95% CI = 1.32-2.51, p < 0.01).

**Table 2:** Univariate and multivariate Cox regression analysis of RNF34 mRNA expression. * RNF34 mRNA expression was dichotomized based on the median. p < 0.05 was considered as significant.

Overall survival						
Parameter	Univariate analysis			Multivariate analysis		
	p-value	Hazard ratio	95% CI	p-value	Hazard ratio	95% CI
RNF34 expression*	<0.001	2.733	1.967 – 3.297	<0.001	2.655	1.639 – 4.302
pT stage	<0.001	1.912	1.624 – 2.251	0.147	1.239	0,927 – 1.656
pN stage	<0.001	3.379	1.794 – 6.365	0.996	1.002	0.477 – 2.104
pM stage	<0.001	4.334	3.178 – 5.912	<0.001	2.628	1.598 – 4.323
Grading	<0.001	2.297	1.877 – 2.811	0.041	1.408	1.014 – 1.957

**Table 3:** Disease-specific survival in patients with ccRCC. Univariate and multivariate Cox regression analysis of RNF34 mRNA expression. * RNF34 mRNA expression was dichotomized based on the median. p < 0.05 was considered as significant. ccRCC: clear cell renal cell carcinoma.

Disease-specific survival						
Parameter	Univariate analysis			Multivariate analysis		
	p-value	Hazard ratio	95% CI	p-value	Hazard ratio	95% CI
RNF34 expression*	<0.001	3.338	2.148 – 5.188	0.004	2.534	1.342 – 4.784
pT stage	<0.001	2.947	2.324 – 3.738	0.002	1.257	0,927 – 2.829
pN stage	<0.001	3.807	1.803 – 8.036	0.286	0.621	0.259 – 1.489
pM stage	<0.001	9.094	6.208 – 13.322	<0.001	4.146	2.339 – 7.349
Grading	<0.001	3.220	2.477 – 4.186	0.161	1.338	0.890 – 2.010

Immunohistochemistry: RNF34 protein expression in ccRCC

**Figure 2:**
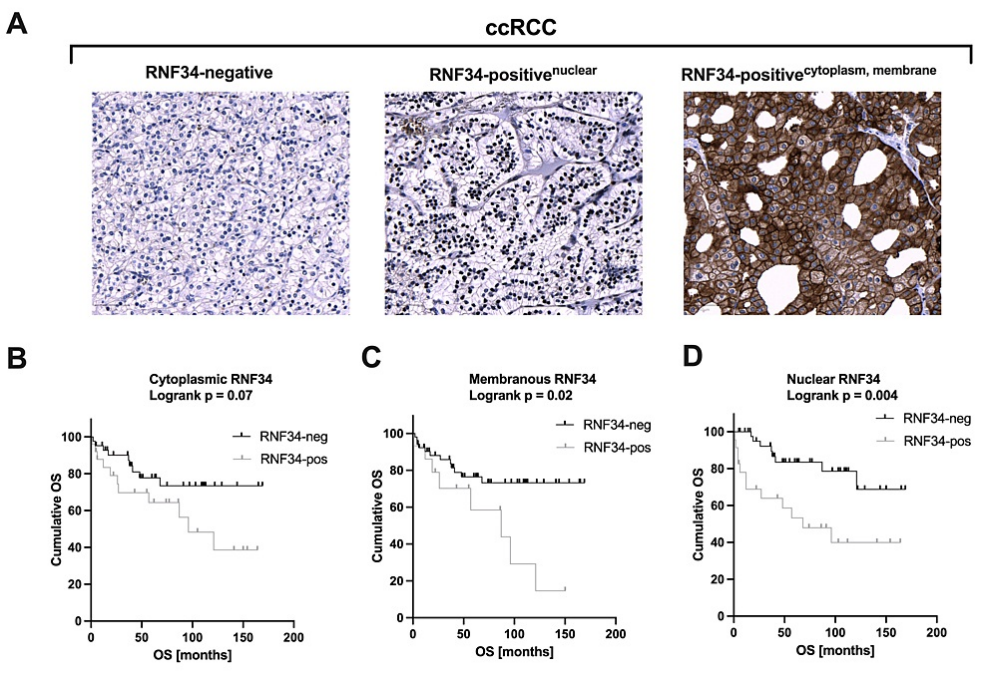
Immunohistochemical staining of RNF34 and Kaplan-Meier analysis of overall survival. (A) Staining patterns of RNF34 protein expression in different compartments: no expression, nuclear, cytoplasmic, and membranous expression; object magnification: 10x, scale bar: 50 µm. Both membranous (C) and nuclear (D) RNF34 protein expression were significantly associated with poor overall survival as determined by Kaplan-Meier estimates (membranous RNF34: log-rank p = 0.02, nuclear RNF34: log-rank p = 0.004). No impact of cytoplasmatic RNF34 expression on survival was evident (log-rank p = 0.07, B). The cut-off describing positive expression was set at ≥1. p < 0.05 was considered as significant. ccRCC: clear cell renal cell carcinoma; OS: overall survival.

To validate the results based on data obtained from the TCGA, the protein expression of RNF34 was analyzed by immunohistochemistry in an independent ccRCC TMA cohort including 109 tumor samples. RNF34 was found to be present in the nucleus, the cytoplasm, and the cell membrane (Figure 2).

In line with the results of the mRNA analysis, RNF34 protein expression is significantly associated with clinicopathological parameters (Table 4). Nuclear RNF34 expression was positively correlated with tumor grade (G1 vs. ≥ G2; p = 0.019) and showed a trend toward significance with positive lymph node status (p = 0.051). A similar result was achieved for cytoplasmic expression; here, expression correlated with the presence of distant (p = 0.026) and lymph node metastasis (p = 0.001) in addition to grading (p = 0.013). However, no significant association with clinicopathological parameters was observed for membranous expression.

**Table 4:** Immunohistochemical analysis of RNF34 protein expression in ccRCC tissues and its association with clinicopathological parameters (chi-square test). p < 0.05 was considered as significant. ccRCC: clear cell renal cell carcinoma.

Parameter	n	RNF34 nuclear			RNF34 membranous			RNF34 cytoplasm		
		Negative	Positive	p-value	Negative	Positive	p-value	Negative	Positive	p-value
pT stage										
pT1/2	63	38	25	0.449	51	12	0.730	42	21	0.280
≥pT3	46	31	15		36	10		26	20	
pN stage										
pN0	100	66	34	0.051	82	18	0.058	67	33	<0.001
pN1	9	3	6		5	4		1	8	
pM stage										
M0	93	62	31	0.079	77	16	0.062	62	31	0.026
M1	16	7	9		10	6		6	10	
Grading										
G1	34	27	7	0.019	30	4	0.14	27	7	0.013
≥G2	75	42	33		57	18		41	34	

Accordingly, patients with RNF34 expression in tumor tissue showed unfavorable clinical outcomes and poor survival. Both membranous and nuclear RNF34 expressions were prognostic of shortened OS, as determined by Kaplan-Meier estimates (membranous RNF34: log-rank p = 0.02; nuclear RNF34: log-rank p = 0.004; Figures 2C, 2D). After accounting for the potential confounders of TNM stage and grading, the multivariate Cox regression analysis also revealed the robust independent prognostic impact of nuclear RNF34 expression on OS (nuclear RNF34: HR = 2.939, 95% CI = 1.09-7.90, p = 0.033; Table 5).

**Table 5:** Overall survival in patients with ccRCC. Univariate and multivariate Cox regression analysis of nuclear RNF34 expression. * RNF34 nuclear expression was dichotomized on positive vs. negative staining (immunohistochemistry). p < 0.05 was considered as significant. ccRCC: clear cell renal cell carcinoma.

Parameter	Univariate analysis			Multivariate analysis		
	p-value	Hazard ratio	95% CI	p-value	Hazard ratio	95% CI
RNF34 nuclear expression*	0.011	3.260	1.309 – 8.117	0.033	2.939	1.093 – 7.903
pT stage	0.271	1.335	0.789 – 2.326	0.747	1.104	0.605 – 2.016
pN stage	<0.001	12.728	4.226 – 38.335	0.164	3.180	0.623 – 16.227
pM stage	<0.001	11.185	3.967 – 31.538	0.010	7.552	1.662 – 35.164
Grading	0.092	2.899	0.839 – 10.017	0.189	2.617	0.624 – 10.980

No significance for a shortened survival of patients with ccRCC was found for cytoplasmic RNF34 expression (log-rank p = 0.07). Additionally, we also observed a correlation between RNF34 protein expression and PFS. Calculated using the Kaplan-Meier estimator, patients with positive membranous or cytoplasmic expression exhibited a shortened PFS (membranous RNF34: log-rank p < 0.01; cytoplasmatic RNF34: log-rank p = 0.012). Based on the results of multivariate Cox regression analysis, elevated membranous RNF34 expression emerged as a significant and independent prognostic factor for reduced PFS in patients diagnosed with ccRCC (HR = 3.139, 95% CI = 1.17-8.46, p = 0.024; Table 6).

**Table 6:** Progression-free survival in patients with ccRCC. Univariate and multivariate Cox regression analysis of membranous RNF34 expression. * RNF34 membranous expression was dichotomized on positive vs. negative staining (immunohistochemistry). p < 0.05 was considered as significant. ccRCC: clear cell renal cell carcinoma.

Parameter	Univariate analysis			Multivariate analysis		
	p-value	Hazard ratio	95% CI	p-value	Hazard ratio	95% CI
RNF34 membranous expression*	0.002	3.855	1.634 – 9.046	0.024	3.139	1.165 – 8.456
pT stage	0.053	1.703	0993 – 2.921	0.109	1.596	0.901 – 2.828
pN stage	0.005	4.947	1.607 – 15.228	0.317	0.424	0.079 – 2.276
pM stage	<0.001	7.048	2.727 – 18.217	0.006	7.443	1.801 – 30.759
Grading	0.100	2.491	0.840 – 7.384	0.033	4.006	1.115 – 14.399

## Discussion

Inhibitors of apoptosis proteins (IAPs) are a protein family involved in the regulation of programmed cell death (apoptosis). They play a key role in controlling cell growth and development. A common feature of all IAPs is the presence of a baculovirus IAP repeat (BIR) domain [12]. So far, several members of the IAPs have been investigated in the context of ccRCC. X-linked apoptosis inhibitor (XIAP), one of the best-evaluated candidates, has been associated with advanced tumor stage and poor histopathological grading as well as shortened survival in previous works [13,14]. Its antiapoptotic properties are mainly linked to its ability to bind to and thereby inactivate initiator and effector caspases [15]. It was stated that overexpression of XIAP decreased the sensitivity of RCC cells to apoptosis and created favorable conditions for tumor cell survival and development. Vice versa loss of XIAP was associated with increased sensitivity to chemotherapy-induced apoptosis in a cell culture model suggesting XIAP as a driver of RCC chemotherapy resistance [14].

In contrast, RNF34 is considered an IAP-like protein that contains a C-terminal RING domain similar to the IAP family. Remarkably, RNF34 differs from the IAP family by containing not the BIR but an FYVE domain, which exhibits a phospholipid-binding activity [16]. The partial plasma membrane localization of the protein is suggested to be mediated by this domain [6]. While IAPs bind to and ubiquitinate caspase-3, caspase-7, and caspase-9 via the BIR domain, RNF34 mediates ubiquitination of caspases 8 and 10 by binding with its caspase-interacting domain [16]. In this manner, IAPs and RNF34 act as antiapoptotic proteins; however, RNF34 is not considered a member of the IAPs but an IAP-like protein due to the aforementioned differences. Furthermore, RNF34 mediates oncogenic properties via degradation of the tumor suppressor p53 and phosphorylated p53 [8]. In addition, RNF34 possesses the ability to hinder cancer cell apoptosis through the regulation of the NOD1 pathway. It directly interacts with NOD1, triggering its ubiquitination and subsequent degradation [17].

RNF34 has been identified previously to play a major role in colorectal cancer (CRC) carcinogenesis [7]. It is upregulated during carcinogenesis and tumor progression in the colorectal adenoma-carcinoma sequence [18]. A recent study based on TCGA data identified RNF34 among the most significant differential expressed genes in CRC [19].

To date, the role of RNF34 in ccRCC has not been studied. Our results point toward an important role of RNF34 in the carcinogenesis of ccRCC: mRNA levels are significantly increased in malignant versus benign kidney tissues. Moreover, high mRNA levels are strongly associated with unfavorable overall survival and are associated with aggressive and advanced tumor stages. Overexpression of RNF34 in CRC cell lines resulted in resistance to 5-fluorouracil (5-FU)-induced apoptosis via nuclear factor-kappa B (NF-kappaB) and enriched BCL-2 and BCL-X expression [19]. In RCC, enriched BCL-2 and BCL-X expression and NF-kappaB activation are also observed and might also contribute to chemotherapy resistance [20-22]. Interestingly, in CRC, RNF34 showed lower expression in advanced cancer stages [19]. Based on this observation, Huo et al. deduce an improved response to therapy with 5-FU in advanced CRC. This is in contrast to our results on ccRCC, which show a higher expression of RNF34 in advanced tumor stages. RNF34 protein expression analysis by immunohistochemical (IHC) staining is consistent with the results based on transcriptome data. Our immunohistochemical analysis revealed the presence of RNF34 in all three cell compartments, i.e., nucleus, cell membrane, and cytoplasm. Yang et al. have already derived conclusions about the localization of RNF34 at the cell membrane and the cytosol. The binding to the cell membrane is mediated by the FYVE domain, which binds to phosphatidylinositol-3-phosphate and its derivates. Cytosolic localization of the protein is most likely due to its interactions with caspases and other cytosolic targets such as p53 [16]. However, the nuclear expression of RNF34 has not yet been elucidated appropriately. Possible explanations include an interaction with the tumor suppressor gene p53 or phosphorylated p53, which are also expressed nuclearly. The nuclear localization of RNF34 expression may also indicate a heretofore unknown protein-DNA interaction, possibly mediated by the zinc finger-like RING finger domain of the protein. Our immunohistochemical examination provides evidence that the prognostic significance of RNF34 depends on the respective targets. While the membranous and nuclear staining is of prognostic relevance, this could not be demonstrated for the cytoplasmic staining. Especially, the prognostic role of nuclear staining suggests that a significant oncogenic function of the protein has not yet been identified.

The study is subject to several limitations. Firstly, the sample size employed in our investigation is constrained (mRNA cohort: n = 533; IHC cohort: n = 109), potentially limiting the extrapolation of our findings to a broader population. Additionally, the retrospective nature of the study poses challenges, as there is a risk of information loss or bias in the collection and interpretation of data. Causal inferences drawn from retrospective data may be subject to limitations. In the context of future research efforts, the implementation of a prospective study design should be pursued. Furthermore, the absence of functional experiments to elucidate direct causal relationships raises questions about the mechanistic underpinnings of the observed associations. Thus, interactions of RNF34 with various proteins, such as p53, could be assumed based on current evidence but are not comprehensively understood, particularly in the context of ccRCC. These limitations collectively underscore the need for cautious interpretation of our results and prompt consideration of these factors in the design and execution of future studies.

## Conclusions

The given work offers corroborative proof of RNF34's role as a prognostic biomarker in ccRCC and points toward a major role of this protein in carcinogenesis. High mRNA and protein expression levels are associated with unfavorable survival and could therefore serve as a surrogate marker for aggressive tumors. Further studies are needed to evaluate to what extent RNF34 could serve as a therapeutic target in patients with ccRCC.
